# pH-Triggered Conformational Switching along the Membrane Insertion Pathway of the Diphtheria Toxin T-Domain

**DOI:** 10.3390/toxins5081362

**Published:** 2013-08-06

**Authors:** Alexey S. Ladokhin

**Affiliations:** Department of Biochemistry and Molecular Biology, The University of Kansas Medical Center, Kansas City, KS 66160, USA; E-Mail: aladokhin@kumc.edu; Tel.: +1-913-588-0489; Fax: +1-913-588-7440.

**Keywords:** acid-induced conformational change, membrane protein insertion, histidine protonation, fluorescence, molecular dynamics, conformational switch

## Abstract

The translocation (T)-domain plays a key role in the action of diphtheria toxin and is responsible for transferring the catalytic domain across the endosomal membrane into the cytosol in response to acidification. Deciphering the molecular mechanism of pH-dependent refolding and membrane insertion of the T-domain, which is considered to be a paradigm for cell entry of other bacterial toxins, reveals general physicochemical principles underlying membrane protein assembly and signaling on membrane interfaces. Structure-function studies along the T-domain insertion pathway have been affected by the presence of multiple conformations at the same time, which hinders the application of high-resolution structural techniques. Here, we review recent progress in structural, functional and thermodynamic studies of the T-domain archived using a combination of site-selective fluorescence labeling with an array of spectroscopic techniques and computer simulations. We also discuss the principles of conformational switching along the insertion pathway revealed by studies of a series of T-domain mutants with substitutions of histidine residues.

## 1. Introduction

**Figure 1:**
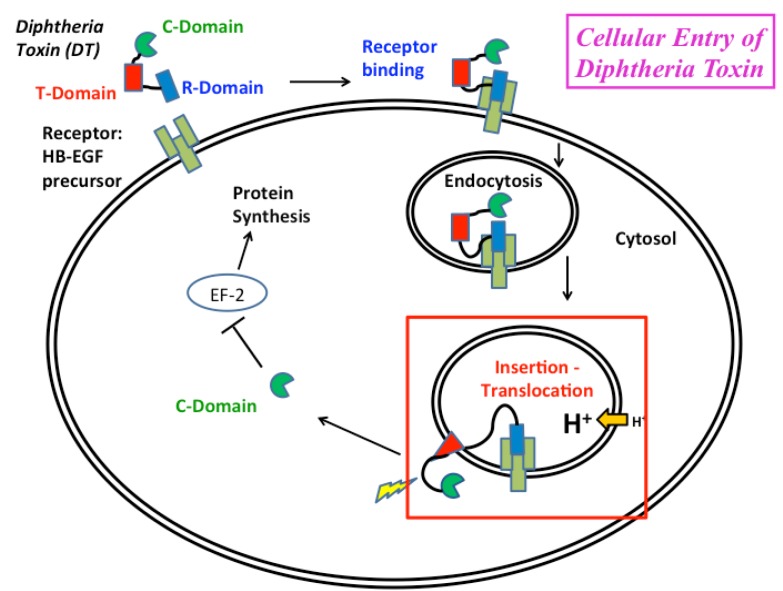
Schematic representation of the endosomal pathway of cellular entry of diphtheria toxin, DT (adapted from [1]). The toxin consists of three domains: receptor-binding (R) domain, responsible for initiating endocytosis by binding to the heparin-binding EGF (epidermal growth factor)-like receptor; translocation (T)-domain; and catalytic (C)-domain, blocking protein synthesis via modification of elongation factor 2. This review is concerned with pH-triggered conformational change of the T-domain resulting in refolding, membrane insertion and translocation of the C-domain (highlighted by the red rectangle).

Diphtheria toxin enters the cell via the endosomal pathway [1], which is shared by many other toxins, including botulinum, tetanus and anthrax [2,3,4,5]. The processes involved in the cellular entry of these toxins are complex and not fully understood. It is clear, however, that they have certain similarities with the entry pathway of diphtheria toxin: they involve receptor-mediated endocytosis followed by endosome acidification and pH-triggered conformational change that results in membrane insertion of the transporting protein and the formation of a pore or a transient passageway through which the toxic enzymatic components enter the cell (Figure 1). In the case of diphtheria toxin, the bridging of the lipid bilayer is achieved through acid-induced refolding and membrane insertion of the translocation (T)-domain. Although T-domain has been a subject of numerous biophysical studies over the years [6,7,8,9,10,11,12,13,14,15,16,17], a consistent picture that would explain its action on a molecular level has yet to emerge. Here, we will review the results of structural and thermodynamic studies of T-domain refolding and membrane insertion obtained in our lab for the past decade.

## 2. Overview of the Insertion Pathway

### 2.1. Summary of Early Studies

**Figure 2:**
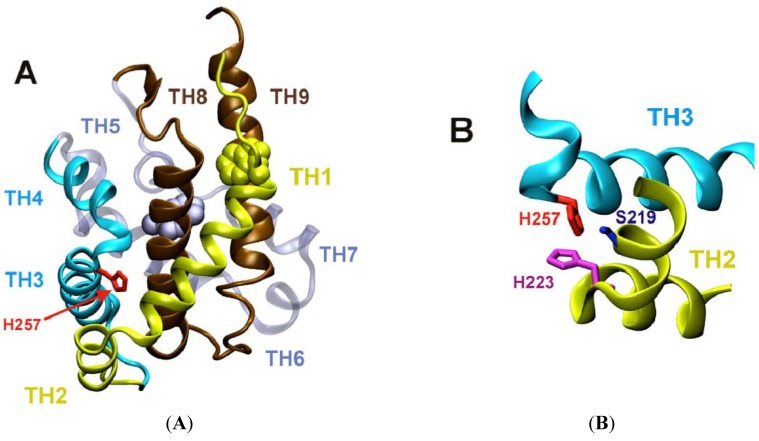
(**A**) Backbone ribbon representation of the crystallographic structure of the T-domain [18]. Histidine 257 (red), critical for pH-triggered refolding [27], is positioned between helices TH1-2 (yellow) and TH3-4 (blue). Other regions of the protein are: consensus membrane insertion domain, TH8-9, in brown and helices TH6-7 in grey. Two tryptophan residues are shown as space-filling models: W206 in yellow and W281 in grey. Lower panel (**B**) represents another view of the region surrounding H257, including H223 (purple), suggested to act as a safety latch preventing premature unfolding by modulating protonation of H257 [28].

**Figure 3:**
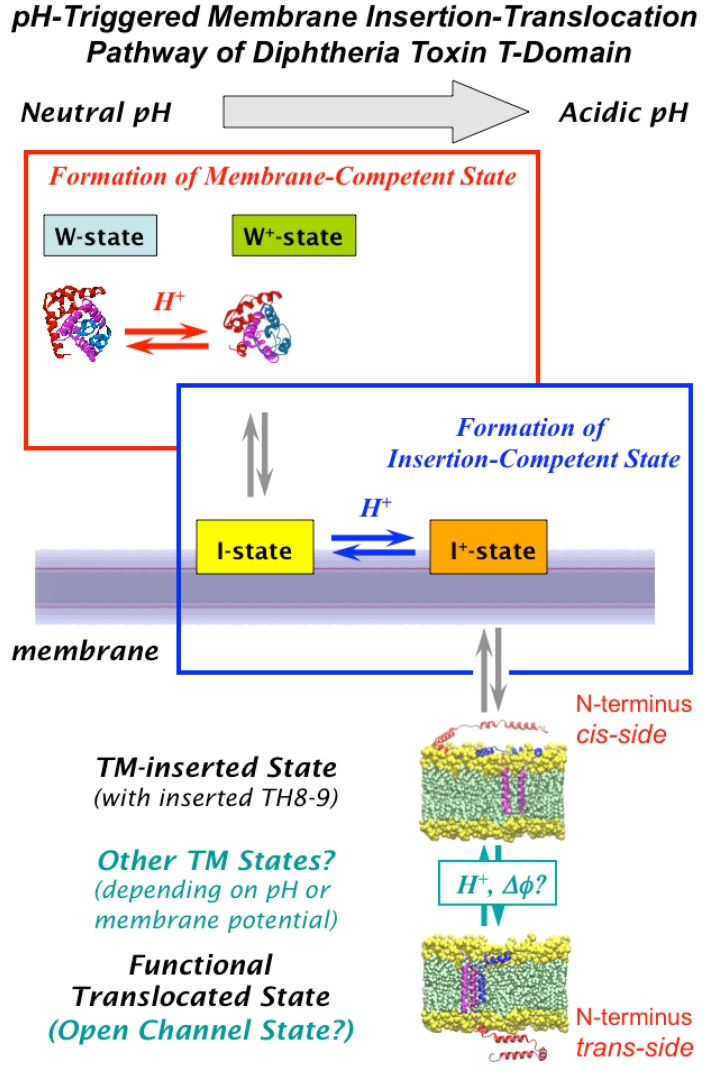
Schematic representation of the pH-dependent membrane insertion pathway of the diphtheria toxin T-domain (modified from [26]). Initial protonation, resulting in conversion of membrane-incompetent W-state to membrane-competent W^+^-state, occurs primarily in the bulk of the solution. In the presence of membranes, this state rapidly associates with the bilayer to form an interfacial intermediate I-state. Subsequent insertion is facilitated by the presence of anionic lipids, which promote the formation of the insertion-competent I^+^-state and decrease the thermodynamic barrier for insertion into the TH8-9 helical hairpin. The two protonation steps responsible for the formation of conformations capable of membrane association (W-to-W^+^ transition, red rectangle) and insertion (I-to-I^+^ transition, blue rectangle) have overlapping pH ranges, suggesting that additional protonation can occur at the same pH value, due to the shift of pKa values of titratable residues after their partitioning into the interfacial zone of the lipid bilayer. While the structure of the functional state of the T-domain on the membrane remains unknown, experimental evidence suggests coexistence of multiple transmembrane (TM)-inserted states, possibly affected by pH and membrane potential (see text and Figure 6 [29]).

The crystallographic structure of diphtheria toxin T-domain in the water-soluble form [18,19] (Figure 2A) provides a starting point for refolding/insertion studies. The protein consists of nine helices of various lengths (TH1-9), eight of which completely surround the most hydrophobic one, TH8. Helices 1 through 4 do not penetrate into the membrane, apparently, and are likely translocated along with the catalytic domain [20,21]. The two proposed models for the fully inserted functionally relevant state are the double dagger model [19] (derived from solution crystallographic structure) and the open-channel state model [9] (derived from numerous measurements of conductivity in planar bilayers [22,23,24]). Supporting evidence from other types of experiments is somewhat contradictory, and the flowing decade-old quote from the authors of the open-channel model still holds true: “by picking and choosing, one can select data from vesicle and cell membrane experiments supporting most of the T-domain topography” [9]. Part of the problem appears to be the difference in the nature of the information obtained by various methods and variations in sample preparation. Nevertheless, both conductivity measurements in planar bilayers [25] and spectroscopic measurements in vesicles [14] indicate that the active form of the T-domain is a monomer. In addition, a number of studies had reported the co-existence of multiple insertion intermediates [11,12,13,14,15,26]. While this conformational lability of the T-domain is not surprising, given the large-scale refolding required for insertion, it certainly complicates the application of high-resolution methods (e.g., X-ray crystallography and NMR) for structure determination of membrane-inserted T-domain. Our goal is to obtain atomistic representation of the T-domain structure along the entire insertion/translocation pathway into and across the lipid bilayer (illustrated by a scheme in Figure 3) and characterize the thermodynamics of the process. Below, we summarize our progress in achieving this task by combining various methods of fluorescence spectroscopy, such as fluorescence correlation spectroscopy, Förster resonance energy transfer and fluorescence lifetime quenching, and computer simulations.

### 2.2. pH-Dependent Formation of Membrane-Competent Form

**Figure 4:**
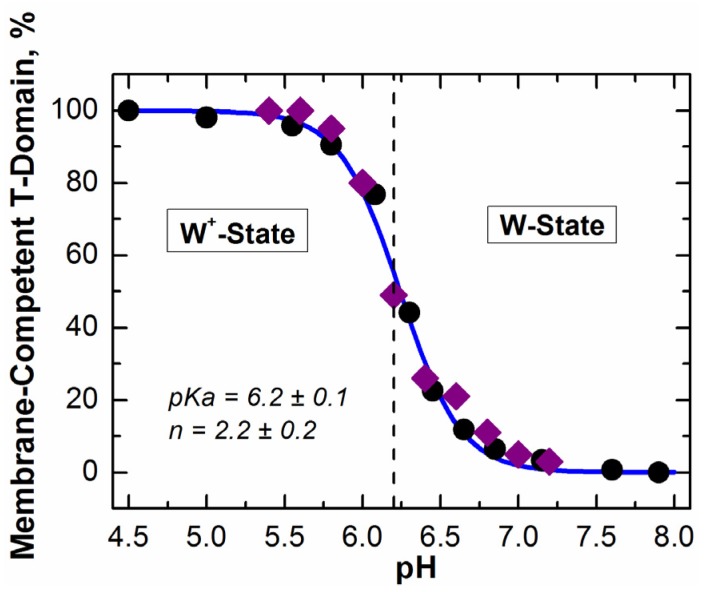
pH-dependent conversion of the T-domain from the soluble W-state into the membrane-competent W^+^-state, identified through the following measurements of membrane binding at lipid saturation [26]: Fluorescence Correlation Spectroscopy-based mobility measurements (diamonds); measurements of FRET (Förster resonance energy transfer) between the donor-labeled T-domain and acceptor-labeled vesicles (circles). The solid line represents the global fit of the combined data [28].

Formation of the membrane-competent form (W^+^-state) of the T-domain is the first step along a complex pathway, leading from a soluble conformation with a known crystallographic structure (W-state), ultimately to membrane-inserted states, for which no high-resolution structural information is available. Initially, this state was identified through membrane binding at lipid saturation [26], and subsequently, its conformation has been characterized via a combination of spectroscopic experiments and all-atom Molecular Dynamics (MD) simulations [28]. pH-dependent transition between the W-state and W^+^-state has a midpoint at pH 6.2 (with a Hill coefficient, *n*, of two) and is over at pH 5.5 (Figure 4), *i.e*., in the pH range associated with early endosomes [30,31,32]. The structural rearrangements during formation of the W^+^-state are subtle, and this state was missed in early studies, which misidentified a molten globule state, formed at pH < 5, as a main membrane-binding species. Extensive microsecond-scale MD simulations performed with the ANTON supercomputer [33,34] reveal that the formation of the W^+^-state, triggered by the protonation of histidine residues, is not accompanied by the loss of structural compactness of the T-domain, while, nevertheless, resulting in substantial molecular rearrangements. A combination of simulation and experiments reveal the partial loss of secondary structure, due to unfolding of helices TH1 and TH2, and the loss of close contact between the *C*- and *N*-terminal segments [28]. The structural changes accompanying the formation of the membrane-competent state ensure an easier exposure of the internal hydrophobic hairpin formed by helices TH8 and TH9, in preparation for its subsequent transmembrane insertion.

### 2.3. Kinetic Insertion Intermediates

Over the years, several research groups have presented compelling evidence for the T-domain adopting multiple conformations on the membrane [10,11,12,13,15], and yet, the kinetics of the transition between those forms has seldom been addressed. Several of those studies used intrinsic tryptophan fluorescence as a primary tool, which makes kinetic measurements difficult to implement and interpret, because of a low signal-to-noise ratio and a sometimes redundant spectroscopic response of tryptophan emission to binding, refolding and insertion. Previously, we have applied site-selective fluorescence labeling of the T-domain in conjunction with several specific spectroscopic approaches to separate the kinetics of binding (by FRET) and insertion (by environment-sensitive probe placed in the middle of TH9 helix) and explicitly demonstrate the existence of the interfacial insertion intermediate [26]. Direct observation of an interfacially refolded kinetic intermediate in the T-domain insertion pathway confirms the importance of understanding the various physicochemical phenomena (e.g., interfacial protonation [35], non-additivity of hydrophobic and electrostatic interactions [36,37] and partitioning-folding coupling [38,39]) that occur on membrane interfaces. This interfacial intermediate can be trapped on the membrane by the use of a low content of anionic lipids [26], which distinguishes theT-domain from other spontaneously inserting proteins, such as annexin B12, in which the interfacial intermediate is observed in membranes with a high anionic lipid content [40,41]. The latter can be explained by the stabilizing Coulombic interactions between anionic lipids and cationic residues present in the translocating segments of annexin. In contrast, in the T-domain, the only cationic residues in the TH8-9 segment are located in the top part of the helical hairpin (H322, H323, H372 and R377) and, thus, will not prevent its insertion. As a matter of fact, placing positive charges on the top of each helix is expected to assist insertion by providing interaction with anionic lipids. Indeed, triple replacement of H322/H323/H372 with either charged or neutral residues was observed to modulate the rate of insertion [42].

The reported non-exponential kinetics of insertion transition [26] clearly indicates the existence of at least a single intermediate populated after the initial binding event (formation of the I-state), but before the final insertion is achieved (formation of the T-state). Similarly to the membrane-competent state, we refer to this intermediate as an insertion-competent state. While the formation of the membrane-competent state (or membrane binding-competent state) leads to the conformation that can bind membrane, the formation of the insertion-competent state leads to the state that can adopt a TM conformation. The formation of this intermediate is both lipid- and pH-dependent, with anionic lipids being essential for its formation (*i.e.*, increasing the population of protein capable of insertion at a given pH), as well as for increasing the overall insertion rate [26]. The mechanism for these effects is not known, although one can reasonably assume that variation in the local concentration of protons near membranes with different contents of anionic lipids can play a certain role. Other explanations involving direct interaction of anionic lipids with the intermediate and insertion-activated transient state should be considered, however.

### 2.4. Insertion Pathway with Two Staggered pH-Dependent Transitions

Various aspects of the pH-triggered bilayer insertion of the T-domain are illustrated using a pathway scheme in Figure 3. The initial protonation step, the formation of membrane-competent form W^+^, occurs in solution and depends little on the properties of the membrane [26]. (This is not always the case for pH-triggered membrane protein insertion—for example, that of annexin B12, which inserts into a TM conformation at low pH in the absence of calcium. In the case of annexin, however, the formation of a membrane-competent state occurs not in the bulk of solution, but on the bilayer interface, and its pH-dependence is modulated by lipid composition via surface potential [41]). The T-domain in this membrane-competent conformation is susceptible to aggregation, but it can be stabilized by fluorinated non-detergent surfactants that act as insertion chaperones [14,43]. Application of such surfactants is essential for equilibrium thermodynamic studies of insertion [17], but is not practical for kinetic studies. In the presence of membranes, the W^+^-state rapidly associates with the bilayer interface (I-state). It is not clear what structural rearrangements are associated with this transition. Final TM insertion requires the formation of the insertion-competent form (I^+^), which is populated in another pH-dependent transition and depends strongly on the fraction of anionic lipids and less on the nature of lipid headgroups [26,29].

**Figure 5:**
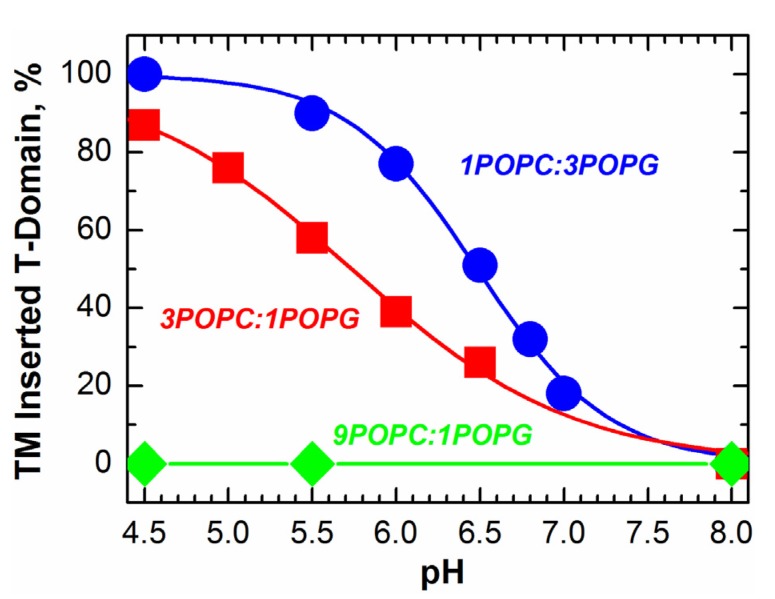
pH-dependent transmembrane (TM) insertion of the T-domain into the vesicles with various lipid compositions measured by fluorescence of the environment-sensitive probe, NBD (*N*-(7-nitro-2-1,3-benzoxadiazol-4-yl), attached to a single cysteine in the middle of TH9 helix [26]. Insertion is promoted by anionic lipids (molar ratios of POPC(palmitoyloleoylphosphatidylcholine)-to-POPG(palmitoyloleoylphosphatidyl­glycerol) three-to-one1 shown in red and one-to-three in blue). No TM insertion is observed when the POPC-to-POPG ratio is nine-to-one (green); even the protein is completely bound to the membrane in the interfacial I-state (Figure 3). This lipid-dependent TM insertion is independently confirmed by topology experiments [26] based on the fluorescence lifetime quenching method [44].

An important aspect of the insertion pathway is that the two pH-dependent transitions, W-to-W^+^ and I-to-I^+^, are not sequential, but staggered, *i.e.*, the second transition starts well before the first one is completed [26] (compare Figure 4 and Figure 5). This implies additional protonation of the T-domain at the same pH to the membrane interface, which can be explained by the change in the pKa of titratable groups responsible for insertion once they are removed from an aqueous environment. The acidic residues, E349, D352 and E362, located in the TH8-9 insertion hairpin, are the likely candidates. Moreover, it is possible that their protonation will be affected by the presence of negative charges on the membrane, which would explain the promotion of insertion by anionic lipids. Quite possibly, the existence of overlapping protonation transitions is an essential feature of all pH-driven membrane protein interactions.

### 2.5. Multitude of TM-Inserted States Conundrum

One of the possible reasons for the absence of a high-resolution structure of the T-domain in the final inserted conformation could be the fact that there is no single conformation in the transmembrane state, but, rather, a collection of states with different folds and topologies. It is clear that one can hardly expect the T-domain to form a regular large pore (for example, one similar to that of anthrax toxin [5]), and it is possible that the molecular species responsible for the physiological function of catalytic domain translocation is formed only transiently. Nevertheless, certain general features of the family of inserted states can be identified. For example, most studies agree that in the inserted state (or states), a hydrophobic helical hairpin, TH8-9, adopts a TM conformation [6,10,26]. The insertion of this consensus domain, however, appears to depend on the exact nature of the sample. The EPR measurements that indicate a TM conformation of these helices [6] are performed using large unilamellar vesicles (LUV) as a membrane system and using a lipid-to-protein ratio of *R*_i_ = 500. Normally, the inserted T-domain is separated from the rest of the sample by centrifugation prior to Electron Paramagnetic Resonance measurements. On the other hand, it has been suggested that efficient insertion requires either a high protein concentration (or low *R*_i_, ~400) or the use of short-chained lipids, such as dimyristoylphosphatidylcholine [10], and can proceed only in small unilamellar vesicles (SUV) [10], but not in LUV [11]. (Unlike larger extruded LUV, sonicated SUV are not equilibrium structures and can result in irregular protein and peptide penetration, as discussed in [45]). In contrast, we were able to utilize the fluorescence lifetime quenching topology method [44] to demonstrate that TH8-9 does adopt a TM conformation in LUV composed of POPC:POPG mixtures, even at *R*_i_ = 3,000, but in a lipid-dependent manner, with anionic lipids greatly favoring the insertion [26]. (It is possible that the low content of anionic lipids in the sample is responsible for the reported conformation of the T-domain with helices parallel to the interface [46]). In addition, our mutagenesis data, discussed in detail below, indicate that insertion of TH8-9 is not necessarily followed by proper insertion of the rest of the protein or translocation of the terminus [42]. It is clear that identifying and characterizing membrane-inserted states constitutes a bottleneck in deciphering the mechanism of action of the T-domain and that progress in this area will require application of new methods and approaches. One of the promising directions of such studies appears to be a utilization of integrated methodologies, combining various spectroscopic techniques with computer simulations.

## 3. Role of Histidine Protonation in Conformational Switching

### 3.1. Mutagenesis Studies

Two groups of residues are expected to undergo protonation in the range of pH relevant to physiological changes inside the endosome: acidic residues (aspartic and glutamic acid), which will lose negative charge upon acidification, and histidines, which will gain a positive charge. Histidine protonation has been implicated in the biological activity of other toxins, including anthrax [47] and aerolysin [48]. It has been suggested that the protonation of the six native histidines of the T-domain makes a favorable thermodynamic contribution to the formation of the interfacial intermediate state of the T-domain [13] and is implicated in the modulation of insertion by anionic lipids [26]. The role of histidines in the action of T-domain has been addressed by Perier *et al*. [16], who studied the membrane interactions of a series of mutants with H-to-F replacements. Such a replacement results in the potential introduction of strong, non-native hydrophobic interactions with the lipid bilayer [49]. In our studies, we have designed an alternative mutagenesis strategy, which is based on comparison of the biophysical and physiological properties of the T-domain, wild type (WT), with those of (a) mutants with neutral, but not hydrophobic residues (H-to-Q replacement) and (b) those with pH-independent positive charge (H-to-R or H-to-K replacements) [27,29,42].

#### 3.1.1. Role of H257 as a Major Component of pH-Dependent Conformational Switch

The effects of systematic replacement (one-by-one and in groups) of all six native histidines of the T-domain with either glutamine or arginine residues on folding in solution was studied by means of circular dichroism (CD) and intrinsic fluorescence [27]. Some replacements (e.g., those of H251) caused pronounced misfolding, while others had only moderate effect on changes of secondary structure. The most intriguing result was obtained with substitutions of H257: a replacement with the neutral glutamine caused little effect at neutral pH, while replacement with the charged arginine caused substantial unfolding. Remarkably, this unfolding was completely reversed by membrane insertion at acidic pH, where CD and fluorescence spectra of H257R mutant regained a WT-like appearance. This behavior is reminiscent of that of intrinsically disordered proteins, with the lipid bilayer playing the role of a ligand, causing gain of structure. Interesting results were also revealed by studies of permeabilization of vesicles loaded with the fluorophore/quencher pair by H257R and H257Q mutants of the T-domain [27]. Whereas both mutants exhibit similar final levels of permeabilization at pH 4.5, the kinetics of release caused by the H257Q mutant is orders of magnitude slower than that of H257R or WT. This indicates that removing the positive charge on H257 significantly affects pH-triggered conformational switching in the T-domain, but does not eliminate it completely, suggesting that such switching is redundant (*i.e*., it can be triggered by multiple residues). Consistent with this mechanism, introducing a pH-independent positive charge at this position is expected to result in an increased activity at neutral pH, which is, indeed, observed for the H257R mutant [27].

The central role of protonation of H257 in destabilizing the folded structure of the T-domain in solution has been confirmed with thermodynamic integration calculations based on a series of MD simulations. The energy penalty for protonation of H257 in the context of the W-state was found to be 6.9 kcal/mole (10.2 kcal/mole, if easily protonatable H223 is already charged), which is the highest among the six histidines [28]. This penalty alone is quite sufficient to overcome the folding free energy of the T-domain, which is on the order of 6–7 kcal/mole. We will further discuss the implications of theoretical predictions of protonation of H223 and H257 based on Poisson-Boltzmann calculations of pKa distributions in the subsequent section.

#### 3.1.2. Role of C-Terminal Histidine Cluster in Membrane Insertion and Translocation

*C*-terminal histidine residues, H322, H323, and H372, have a peculiar location, flanking the consensus insertion domain, TH8-9. The replacement of the three *C*-terminal histidine residues in triple-R or triple-Q mutants prevents effective translocation of the N-terminus, while introduction of these mutations in the full-length toxin results in the decrease of its potency [42]. In the context of isolated T-domain, these mutations cause loss of characteristic conductance in planar bilayers. Surprisingly, these mutations do not affect general folding in solution, protein interaction with the membranes and insertion of the consensus transmembrane helical hairpin, TH8-9 [42]. This indicates the existence of multiple inserted states of the T-domain with various membrane topologies (Figure 3, lower panel). Thus, the C-terminal histidine residues are critical for the transition from the inserted intermediate state to the open-channel state in the insertion/translocation pathway of the T-domain. Recently, we have demonstrated that these effects are mainly due to the replacement of H322, although other histidines also influence the insertion pathway [29].

**Figure 6:**
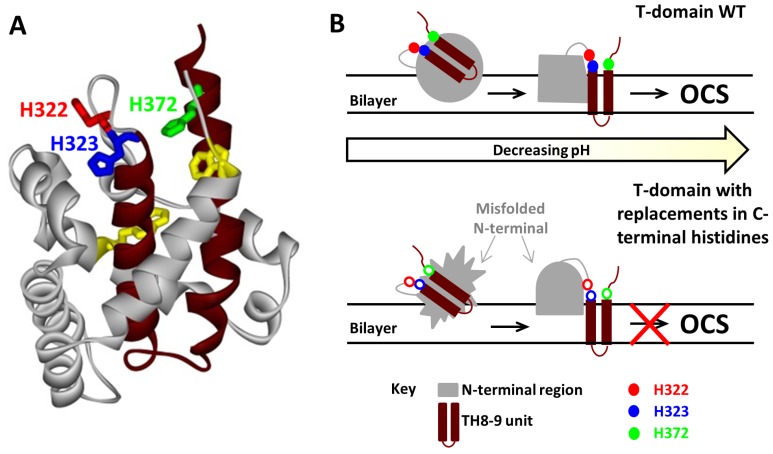
Role of C-terminal histidines in modulating membrane-insertion pathway of the T-domain [29,42]. (**A**) *C*-terminal histidines, H322, H323 and H372, are located on top of the insertion unit comprising a helical hairpin TH8-9 (highlighted in brown) in the crystal structure of the soluble form of the diphtheria toxin T-domain. Tryptophan residues W206 and W281 are shown in yellow, and the rest of the protein is shown in grey; (**B**) Schematic representation of the differences in the insertion process of the WT T-domain and its mutants. Top (WT T-domain): upon initial destabilization of the WT T-domain and its association with the lipid bilayer, the *N*-terminal region of the protein adopts a conformation that leads to the insertion of the TH8-9 unit into the bilayer. The *N*-terminal region refolds to form the open channel state (OCS). Bottom (mutants with *C*-terminal histidine replacements): membrane interaction of these mutants results in a different conformation from that of the WT, specifically in the more exposed *N*-terminal part, as revealed by a red-shifted fluorescence. While the initial insertion of TH8-9 is not compromised by the mutations [42], the replacement of *C*-terminal histidines, especially that of H322, affects efficient folding of the T-domain into the OCS [29].

We illustrate the role of *C*-terminal histidines in the scheme summarizing membrane insertion of the WT T-domain and the mutants carrying substitutions of the *C*-terminal histidines (Figure 6). Upon initial formation of the membrane-competent state and binding to the membrane, the process continues through the insertion of TH8-9 into the bilayer and the subsequent refolding of the rest of the protein, until reaching the open-channel state [26]. It is proposed that the *C*-terminal histidines are involved in guiding the conformation of the *N*-terminal region through productive folding intermediate states towards the Open Channel State (OCS). There is no high-resolution structure of the OCS available (or that of any membrane-associated intermediate); however, the electrophysiological data are consistent with helices TH8, TH9 and TH5 adopting a transmembrane conformation [9]. When *C*-terminal histidines are replaced, the protein still undergoes a proper pH-dependent destabilization in solution, binds to membranes [29] and inserts a TH8-9 helical hairpin [42] similar to that of the WT. Histidine replacement, however, leads to the formation of a non-productive intermediate that is detected by spectral measurements of intrinsic fluorescence, indicating greater exposure of W206 and W281 to the aqueous phase at pH values of ~6–6.5. The replacement of H322 appears to be particularly damaging, as the corresponding mutants tend to misfold and, possibly, aggregate on the membrane, dramatically reducing the number of properly folded and functional channels. Interestingly, the replacement of H322 with the charged or neutral residue has a similar effect on the folding pathway, which is different from replacements of another critical residue, H257, involved in destabilization of the folded structure in solution [27] discussed above.

### 3.2. Computer Simulation Studies

**Figure 7:**
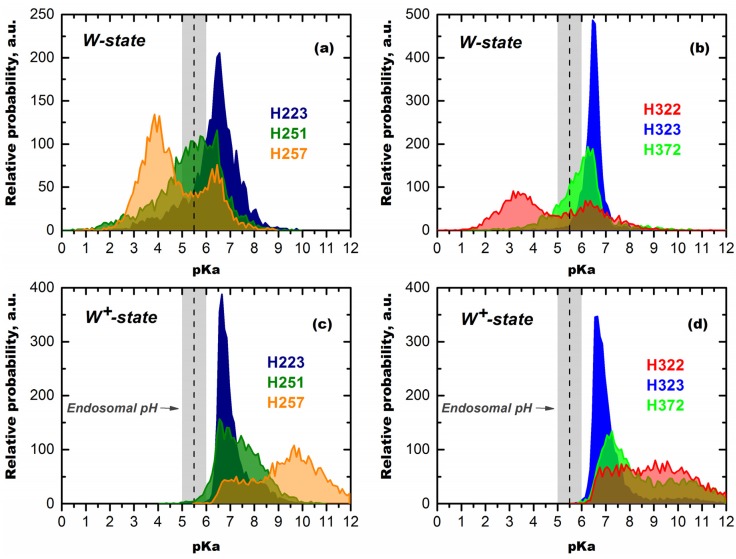
pKa distributions for *N*-terminal (**a**,**c**) and *C*-terminal (**b**,**d**) histidine residues of the T-domain calculated in Poisson-Boltzmann approximation from Molecular Dynamics (MD) traces for the membrane-incompetent W-state (a,b) and the membrane-competent W^+^-state (c,d) (data for the entire MD trace are published in [28]). Remarkably, the only two residues with bimodal distribution of pKa are those that were shown to be critical to refolding in solution (H257) and to guiding the insertion from the membrane interface (H322) by mutagenesis studies [27,29]. Note that under conditions of endosomal pH, all six histidines are predicted to be protonated in the W^+^-state. Coupling of histidine protonation to the conformational change results in a complete conversion of the T-domain to the membrane-competent state by pH 5.5, which is observed experimentally (Figure 4).

Cellular entry of DT starts with receptor-mediated endocytosis [1], but the critical step occurs inside the endosome, resulting in bridging the membrane of the compartment by the T-domain, followed by translocation of the catalytic domain. How do the above-discussed biophysical studies performed *in vitro* or *in silico* relate to the process of cellular entry, and what can we learn from them about molecular mechanism of *in vivo* action of the T-domain? The initial states on the insertion pathway (Figure 3) can be a map of cellular entry (Figure 1) in the following way: the membrane-incompetent W-state corresponds to the state outside the cell, while the protonated membrane-competent W^+^-state corresponds to the state inside the endosome. The pH range of 5.5–6.5 measured for the W-to-W^+^ *in vitro* (Figure 4) corresponds well to the pH range in early endosomes [30,31,32]. Biophysical experiments and MD simulations allow us to take a look at how the T-domain prepares to make cellular entry with molecular resolution. Recent results demonstrate with atomistic detail how protonation of histidines triggers a conformational change that prepares the T-domain for membrane binding and insertion (e.g., breakage of long TH-1 helix and exposure of the TH8-9 consensus insertion domain) [28]. In addition to these structural rearrangements, our calculations reveal important thermodynamic implications of histidine protonation for modulating cellular action of the T-domain. We illustrate these findings in Figure 7, which presents the results of Poisson-Boltzmann calculation of pKa values for all six histidines of the diphtheria toxin T-domain, both in W- and W^+^-states.

The advantage of long microsecond-scale MD simulations is that they allow one to explore in great detail the distribution of conformational states and characterize their thermodynamic properties, such as the pKas of titratable groups. As a result, rather than analyzing a single average pKa available for static crystallographic structure, we have at our disposal entire distributions (Figure 7). It is remarkable that the only two histidine residues to exhibit a double-headed distribution of pKas, namely H257 and H322 [28], are those that were identified through mutagenesis as being critical for refolding in solution [27] and on membrane interface [29]. We hypothesize that the bimodal distribution of pKas is a hallmark of residues involved in pH-triggered conformational switching, as it allows it to become protonated via a high-pKa mode, but perturbs the structure via a low-pKa mode.

### Mechanisms of pH-Trigger and Safety Latch Suggested by MD Simulations

In the W-state, H223 exhibits a unimodal pKa distribution with a maximum at pKa 6.5 (Figure 7a), which makes it susceptible to protonation, even before endosomal encapsulation of the T-domain. In contrast, H257 has a much lower average pKa of 4.7, distributed at two peaks centered at pH 4 and 6.3. The distribution for H257 is shifted even further to acidic pH by approximately 1.5 units, when calculation is repeated, assuming H223 is already protonated (not shown). These differences are consistent with the thermodynamic integration calculations discussed above, which suggest that protonation of H257 in the context of the W-state is very costly energetically, especially when H223 is protonated [28]. This does not mean, however, that one needs to reach a pH below four to protonate H257 in the cell, because the T-domain undergoes a protonation-coupled refolding to the W^+^-state. To illustrate the concept of this linked equilibrium, consider a particular T-domain molecule that adopts a conformation with a pKa of 5.5 for H257 and, thus, has a 50% probability of a proton on this histidine at pH 5.5. The molecule can remain in this conformation and, eventually, lose a proton or go to another conformation in the W-state ensemble without major structural rearrangements. Alternatively, it can undergo a proton-triggered conformational change leading to the formation of a membrane-competent W^+^-state, in which the probability of remaining protonated at pH 5.5 is 100% (Figure 7). In accordance with the Le Chatelier principle, this coupling will result in complete transfer of the entire population toward the protonated W^+^-state at pH 5.5.

The pKa distributions calculated for the membrane-competent state (Figure 7c,d) indicate that all histidine residues will remain protonated in the endosome. As described above, the implications of this coupling of protonation and conformational change are such that, even at pH 5.5, all molecules of the T-domain will undergo a transition to the W^+^-state, which is indeed observed experimentally (Figure 4). According to the pKa calculations, this transition would have started before the endosomal internalization, if it were not for the effects of H223 lowering the pKa distribution of H257 [28]. Therefore, we suggest that easily protonatable H223 acts as a safety latch for preventing the triggering of the conformational change in the T-domain prematurely. The premature refolding of the T-domain outside the endosomal compartment would be non-productive for the following reasons: (1) the pH is not right for the subsequent states of the insertion pathway and (2) the catalytic domain has not yet undergone an acid-induced destabilization and is not ready to be translocated into the cytosol. We hypothesize that this may be important physiologically, because, otherwise, the protonation of H257 would have caused substantial unfolding before the endosomal compartment is reached and would trigger a non-productive interaction with the membrane at an early stage of the insertion pathway. Thus, H223 can be compared to a safety device, which reduces protonation of the crucial H257 by further shifting its pKa and holding it in a state resembling a loaded spring, until the protein is poised for translocation in the endosomal compartment. Once acidification of an endosome lowers pH sufficiently for the protonation of H257 to occur, the safety latch can no longer hold, and the spring is released, causing the conformational change that results in formation of the membrane-competent state, membrane insertion and translocation.

## 4. Perspectives and Applications

The Diphtheria toxin T-domain has been shown to implement its function―translocation of the catalytic domain across the endosomal membrane under acidic conditions―by itself, without the help of any additional protein component [20]. It has also been suggested that it assists other partially unfolded proteins across the lipid bilayer [50], indicating a general, rather than specific translocation pathway. Recently, this membrane-translocating ability of the T-domain has been utilized to improve cellular delivery of poly(ethylenimine)-based vectors during gene transfection [51]. Diphtheria toxin has been utilized as a prospective anti-cancer agent for the targeted delivery of cytotoxic therapy to cancer cells [52,53,54,55,56,57,58,59,60,61,62,63,64,65]. Normally, the targeting is achieved by deleting the cell receptor-binding R-domain and combining the remaining portion (containing T- and C-domains) with proteins that selectively bind to the surface of cancer cells (one such fusion protein, which contains human interleukin-2 and truncated diphtheria toxin, is approved for use in cutaneous T-cell lymphoma [54,59,60]). While it has been assumed that “receptorless” toxin cannot bind to and kill cells, a recent study demonstrated that recombinant DT385 with a deleted R-domain is, in fact, cytotoxic to a variety of cancer cell lines [52]. Because cancerous cells are known to produce a slightly acidic environment, it is likely that the targeting of “receptorless” toxin is assured by pH-triggered membrane insertion of the T-domain in a fashion similar to that of the pHLIP peptide [66,67]. Understanding the molecular mechanism of T-domain action will influence our ability to rationally design drug delivery systems based on pH-dependent conformational switching.

Biophysical studies of the pH-triggered action of the diphtheria toxin T-domain are expected to impact not only the field of cellular entry of toxins or targeted cellular delivery of therapy, but would also advance our understanding of general physicochemical principles underlying conformational switching in proteins. For example, a number of proteins from the Bcl-2 family, carrying out both pro-apoptotic and anti-apoptotic functions, have been demonstrated to have a solution fold dominated by a hairpin composed of long hydrophobic helices similar to those of the diphtheria toxin T-domain [68,69]. Additionally, similar to the T-domain, they have been shown to form ion channels in artificial bilayers [70]. Although it is not clear exactly how these proteins modulate the apoptotic response, a change in membrane topology has been suggested to play a role [71]. The models proposed for their membrane insertion are almost exclusively based on data generated for membrane insertion of the T-domain. Notably, these models have not been tested experimentally and are based on structural similarities of the solution fold, rather than any thermodynamic analysis of membrane-binding propensities. Deciphering the physicochemical rules governing interactions of the diphtheria toxin T-domain with membranes of various lipid compositions will help generate testable hypotheses of the mode of interaction of the Bcl-2 proteins with the outer mitochondrial membrane during apoptosis. 
